# Fanconi Anemia Core Complex Gene Promoters Harbor Conserved Transcription Regulatory Elements

**DOI:** 10.1371/journal.pone.0022911

**Published:** 2011-08-03

**Authors:** Daniel Meier, Detlev Schindler

**Affiliations:** Department of Human Genetics, University of Wurzburg, Wurzburg, Germany; Southern Illinois University, United States of America

## Abstract

The Fanconi anemia (FA) gene family is a recent addition to the complex network of proteins that respond to and repair certain types of DNA damage in the human genome. Since little is known about the regulation of this novel group of genes at the DNA level, we characterized the promoters of the eight genes (*FANCA*, *B*, *C*, *E*, *F*, *G*, *L* and *M*) that compose the FA core complex. The promoters of these genes show the characteristic attributes of housekeeping genes, such as a high GC content and CpG islands, a lack of TATA boxes and a low conservation. The promoters functioned in a monodirectional way and were, in their most active regions, comparable in strength to the SV40 promoter in our reporter plasmids. They were also marked by a distinctive transcriptional start site (TSS). In the 5′ region of each promoter, we identified a region that was able to negatively regulate the promoter activity in HeLa and HEK 293 cells in isolation. The central and 3′ regions of the promoter sequences harbor binding sites for several common and rare transcription factors, including STAT, SMAD, E2F, AP1 and YY1, which indicates that there may be cross-connections to several established regulatory pathways. Electrophoretic mobility shift assays and siRNA experiments confirmed the shared regulatory responses between the prominent members of the TGF-β and JAK/STAT pathways and members of the FA core complex. Although the promoters are not well conserved, they share region and sequence specific regulatory motifs and transcription factor binding sites (TBFs), and we identified a bi-partite nature to these promoters. These results support a hypothesis based on the co-evolution of the FA core complex genes that was expanded to include their promoters.

## Introduction

The Fanconi anemia (FA; MIM #227650) family of genes is an important component of a multi-member DNA damage defense network that protects the human genome from the detrimental consequences of interstrand DNA crosslinks and stalled replication forks [1]. Fifteen complementation groups and the corresponding genes have been identified to date [2], [3]. To drive the FA/BRCA pathway, eight of the FA proteins, FANCA, -B, -C, -E, -F, -G, -L and -M (NM_000135, NM_001018113, NM_000136, NM_021922, NM_022725, NM_004629, NM_018062, and NM_020937, respectively), and other facultative components, such as FAAP100, FAAP24, MHF1 and MHF2, assemble into a nuclear complex [4], [5], [6], [7], [8], [9]. Possessing E3 ligase activity, the FA core complex monoubiquitinates and activates the downstream FA ID complex, which consists of FANCD2 and FANCI [10]. Some data have suggested an equimolar ratio of the core complex molecules [8], [11], [12], but apart from the phosphorylation of certain components in response to DNA damage (via ATR/Chk1 kinases), little is known about the regulation of the individual genes and the products that make up the core complex. With the exception of a single report describing the 5′-UTR of FANCC, the putative promoter regions of the FA core complex genes have not been characterized [13]. The genes encoding the core complex proteins are located on different chromosomes, and the resulting proteins vary greatly in size [10]. By employing in silico methods, we identified the putative promoter regions as 5′ sequence intervals with low degrees of conservation among vertebrates. Using a standard dual luciferase assay, we were able to assign the strongest level of activity (corresponding to the SV40 promoter) to the middle portion of our promoter constructs. We then looked for the presence or absence of key regulatory motifs and characterized the distribution and conservation of transcription factor binding sites throughout the respective promoter regions [14], [15]. Using electrophoretic mobility shift assays (EMSAs), we investigated the binding affinity of two prominent families of transcription factors (STAT and SMAD) to the FA promoter sequences. Because TGF-β signaling may be defective in FA, we investigated the role of SMAD4 (known as the “common SMAD”) within the context of FA core complex gene regulation. In addition, SMAD1, STAT1 and STAT4 were studied in gene knockdown experiments to determine the potential regulatory correlations between these factors and the promoters of the FA core complex genes.

## Results

### Determination of the transcriptional start sites in FA core complex genes

The TSS information was derived from the database of transcriptional start sites (DBTSS), where experimentally confirmed TSSs are annotated [16]. The FA core complex genes proved to be uniformly characterized by a single major TSS, surrounded by weaker TSSs. This resulted in a relatively broad distribution and a single dominant peak (PB) [17]. The TSSs were generally represented by an adenosine. For genes with multiple putative start sites, we selected the TSS that was most frequently used *in vivo*.

### Identification of the putative FA core complex gene promoters

The sequence information from the FA core complex genes and their flanking sequences were used as guides for the primer design, and several fragments were cloned from human genomic DNA. The identities of the genomic DNA fragments were determined by sequencing. We used three sets of constructs (L1, L2 and L3) that were designated according to their different lengths. The first construct (L1) contained the longest sequence, which was approximately 1 kb upstream of the TSS, and this is a typical length for several promoters that have been described in the literature [18]. The second region (L2) was 402–569 bp in length, and it extended from the most proximal part of the putative promoter to the middle region. The shortest portion (L3) covered the region 186 to 250 bp immediately upstream of the TSS (Table 1).

**Table 1:** Promoter data of the FA core complex genes.

		Promoter region			size			Region relative to the TSS		
gene	chromosome	start	end	strand	L1	L2	L3	L1	L2	L3
		hg 19								
**FANCA**	16	89883006	89884049	(−)	1044 bp	425 bp	219 bp	−1002/+42	−383/+42	−177/+42
**FANCB**	X	14891150	14892204	(−)	1055 bp	436 bp	250 bp	−1021/+34	−402/+34	−216/+34
**FANCC**	9	98079252	98080351	(−)	1099 bp	402 bp	186 bp	−1098/+1	−401/+1	−185/+1
**FANCE**	6	35419104	35420181	(+)	1078 bp	410 bp	225 bp	−1034/+44	−366/+44	−181/+44
**FANCF**	11	22647334	22648403	(−)	1069 bp	415 bp	232 bp	−1046/+23	−392/+23	−209/+23
**FANGG**	9	35079995	35081083	(−)	1089 bp	569 bp	193 bp	−1071/+18	−551/+18	−178/+18
**FANCL**	2	58468480	58469550	(−)	1071 bp	466 bp	223 bp	−1065/+6	−460/+6	−217/+6
**FANCM**	14	45604137	45605214	(+)	1077 bp	456 bp	234 bp	−1012/+65	−391/+65	−169/+65

**Figure 1:**
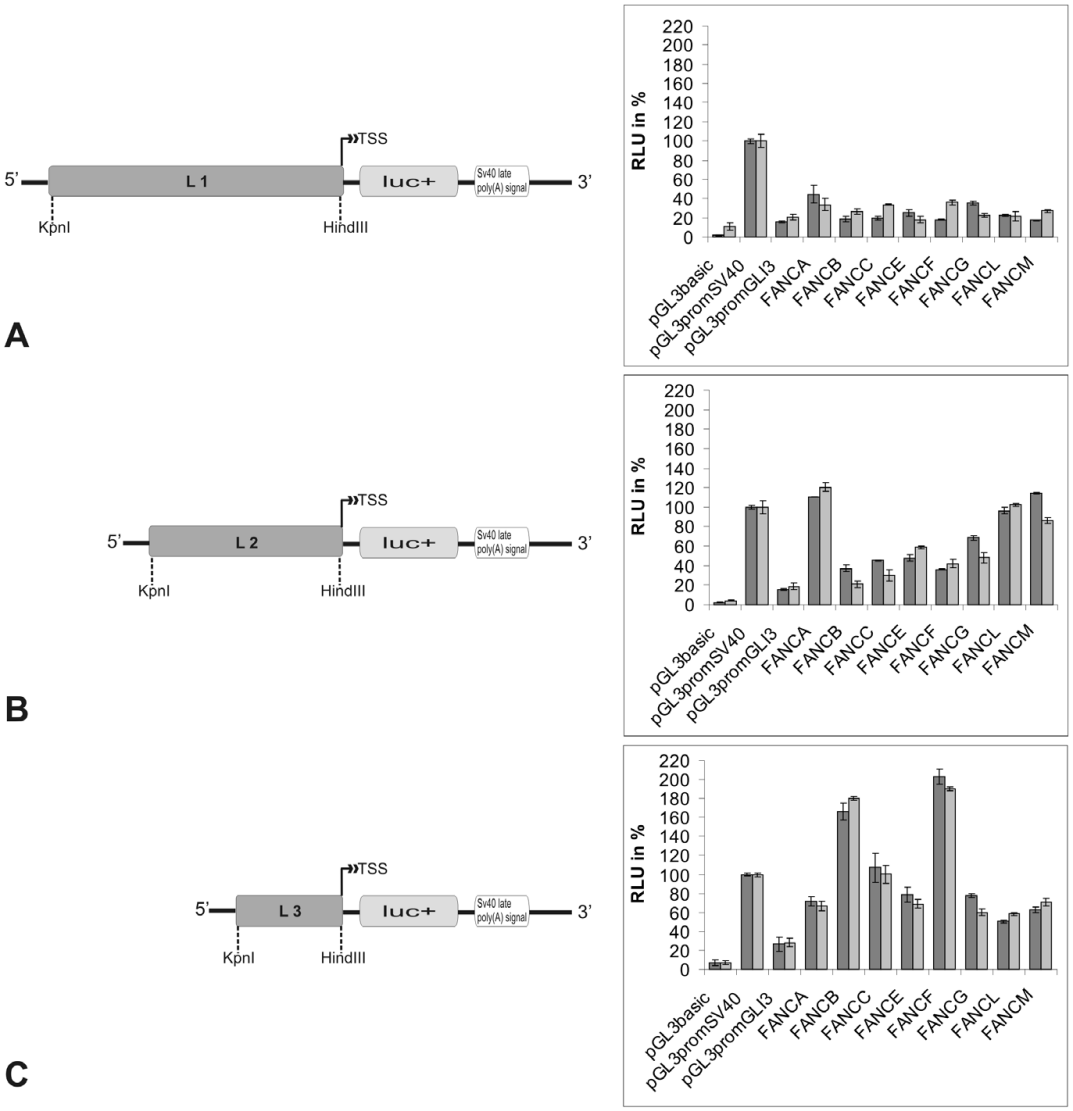
A schematic representation of different FA gene promoter constructs in pGL3 and their measured activities. (**A**) The longest insert (L1) covers the entire promoter region (∼1 kb) upstream of the TSS. (**B**) L2 is a smaller insert of ∼500 bp. (**C**) L3 is the smallest insert and is ∼220 bp upstream of the TSS. In all the samples, the positive control (pGL3 SV40) was set as 100%. The *GLI3* promoter served as the reference for a weak promoter. The results for the HeLa cells are in dark gray, and the results for the HEK 293 cells are in light gray.

The activities of the fragments were examined in a transient transfection experiment using firefly and *renilla* luciferase constructs in HeLa and HEK 293 cells. All of the 5′-flanking regions of the FA core complex genes had significant promoter activities in the transiently transfected HeLa and HEK293 cells (Fig. 1, A–C).

### The FA core complex gene promoters show characteristics of housekeeping gene promoters

The GC content of the promoters was about 70% and higher than the average values for the whole genome. A high number of CpG islands, low conservation through different species and the lack of TATA boxes are characteristics of housekeeping gene promoters [19].

### Differential activities within the FA core complex gene promoters

The L1 to L3 series of reporter plasmids contained different lengths of the FA core complex 5′-flanking region (ranging from 1099 bp to 186 bp) and were upstream of the firefly luciferase gene. To determine the region in these promoters that was required for maximal activity, they were transiently transfected into HeLa and HEK 293 cells. To validate our results, we compared the luciferase activities obtained with the FA gene promoters to those obtained with two known promoter sequences. We tested the SV40 promoter as a strong promoter that was inserted into the pGL SV40 plasmid, and we used the minimal promoter of the human GLI3 gene (NM_000168; Greig cephalopolysyndactyly syndrome; MIM #175700) as an example of a relatively weak promoter [20].

The L1 region accounted for 20% to 50% of the activity of the SV40 promoter (Fig. 1A). A high activity was consistently observed with the L2 region (40% to 115%; Fig. 1B); however, the mean value of the L3 region showed even greater activity (50% to 203%; Fig. 1C). Nevertheless, occasionally some L2 regions show a higher activity than their L3 equivalent (FANCA, -L and -M). The activities of both L2 and L3 were comparable to the SV40 promoter activity. These results showed that the strongest activities of the FA core complex gene promoters were exerted by those neighboring the TSS and that promoter activity decreased as the distance upstream of the TSS increased.

In terms of single FA genes, the *FANCA-* and *FANCM-*derived L2 constructs showed 20% higher activity than the SV40 promoter (Fig. 1B). In contrast, the L2 FANCF promoter construct, which showed the weakest promoter activity of all of the core complex genes, displayed twice the activity of the GLI3 promoter and half of the activity of the SV40 promoter. The L3 portion of the *FANCF* promoter showed the highest activity, which was twice the activity of the SV40 promoter (Fig. 1C).

**Figure 2:**
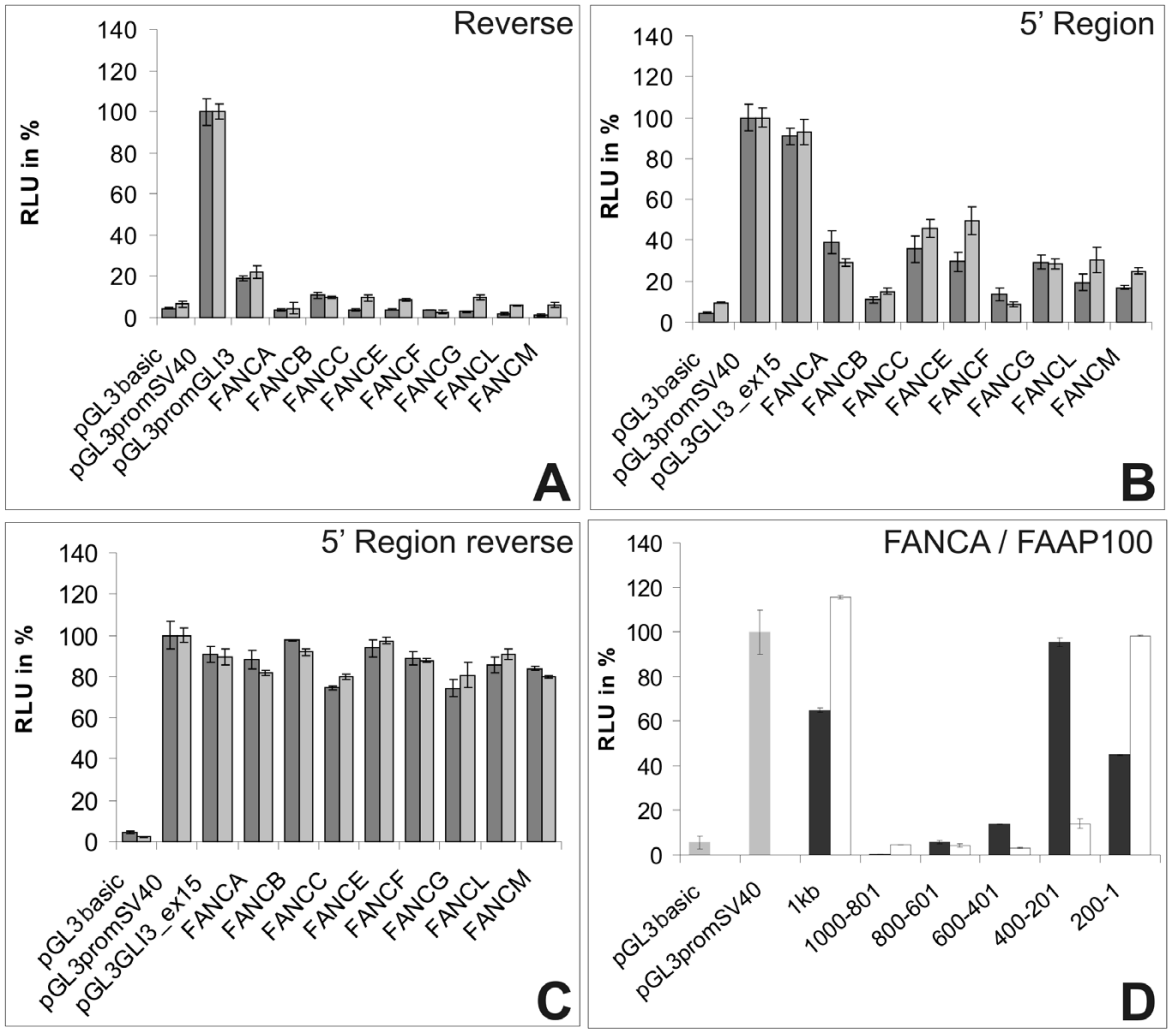
Results of the dual luciferase assays for additional constructs. (**A**) Results for the reverse complementary region (L2). The activity was strongly reduced in both cell lines. (**B**) The region extending from the 5′ end of the entire promoter region to the 5′ end of the L2 region was cloned into the pGL3 SV40 vector. It displayed low promoter activity. (**C**) The same region in (B) but cloned in a reverse complementary orientation. (**D**) The differential activity in *FAAP10*0, compared to the *FANCA* promoter. The results for *FAAP100* are in white, and the results for *FANCA* are in black. The experiment was performed in HEK293 cells.

A feature that was common to all the cloned promoter fragments was their monodirectional activity. This was determined by cloning the L2 promoter fragments in reverse complementary orientation into the pGL3 basic plasmid. With the exception of the *FANCB* promoter, all the other constructs showed little or no activity in the dual luciferase assay in the reverse orientation (Fig. 2A).

### Antagonistic FA promoter activity

To further characterize the activity within the 5′ portions of the FA core complex gene promoters, we amplified the regions from the 5′ end of L1 to the 5′ end of L2. One was amplified in the sense direction and one in the reverse-complement orientation, and the products were cloned into the pGL3 SV40 vector (Figs. 2B and C). As a control, we cloned a sequence extending exactly 600 bp upstream of exon 15 in the *GLI3* gene in the same manner to rule out any site-specific, random repression-like effects due to insertions upstream of the SV40 promoter. The inserts that were cloned in a strand-specific manner differed greatly and this suggests a direction-specific inhibition. The constructs from the *FANCA*, *FANCC* and *FANCE* promoters reduced the firefly luciferase activity to less than 40% of the expression level of the SV40 promoter alone (Fig. 2B). The constructs of the *FANCB*, *FANCF*, *FANCL* and *FANCM* promoters showed even lower activities (5% to 30%) compared to the SV40 promoter alone, which was consistent with the idea that all of these sequences may provide negative activity. The effect of the reverse complement inserted sequences was marginal in both HeLa and HEK293 cells, with a luciferase activity comparable to the control (74% to 98% of the original activity). Our results suggest that the regions upstream of the 5′ end of the L2 sequences act as strand specific silencing elements or silencers of transcription initiation. In all our experiments we did not observe a significant or systematic difference in the results between the HeLa and HEK293 cell lines in their normal or reverse orientations.

### The FAAP100 promoter

FAAP100 (NM_025161) is a recently described member of the core complex and the FANCB-FANCL-FAAP100 subcomplex [5]. FAAP100 is not yet designated as an authentic FA protein because patients with mutations in this gene have not been identified to date. Therefore, FAAP100 was not included in all of our analyses. However, to characterize the *FAAP100* promoter and determine similarities, we divided the putative promoter region (approximately 1 kb) into five segments with lengths of approximately 200 bp. We then cloned the corresponding PCR products into the pGL3 basic plasmid. We measured each of these fragments using the dual luciferase assay in comparison to *FANCA* in the HEK293 line. We did not detect any significant activity in the 5′ region of the promoter sequence of *FAAP100* within −1000 to −601 nt of the TSS, which was divided into 200 bp fragments (Fig. 2D). The section from −600 to −401 nt showed weak promoter activity of no more than 15% of the SV40 promoter activity. The highest level of activity (approaching 95% of the SV40 promoter) was observed in the region from −400 to −201 nt. The final segment (−200 to +1 nt) showed intermediate activity, which was 45% of the SV40 promoter activity. These results indicate that the *FAAP100* promoter has an activity pattern similar to the patterns observed for the other core complex genes, as shown in detail for the *FANCA* promoter.

### Conserved DNA motifs in the *FANC* promoters

**Figure 3:**
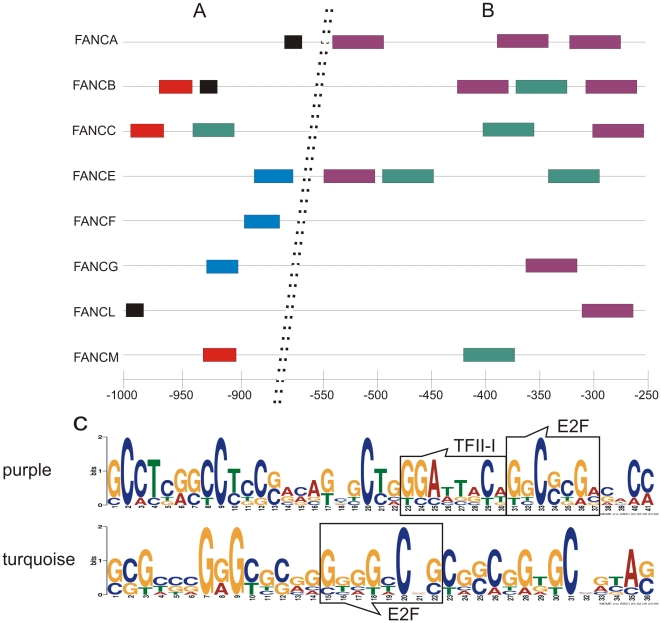
The distribution of conserved DNA motif blocks within the FA core complex gene promoters. (**A**) The 5′- region with a seemingly random distribution of three different sequence motifs. Each motif occurred no more than once per gene. (**B**) Within the region extending from −250 to −550, two sequence motifs (purple and green bars) are present repeatedly or combined in most of the tested FA genes. (**C**) The DNA sequences of the two motifs. The degree of nucleotide conservation is indicated by the height of the respective letters. The purple color represents the upper sequence, and the green color represents the lower sequence.

With the help of the Multiple Em for Motif Elicitation (MEME) software, we identified several DNA sequence motifs that were present in nearly all of the promoter regions of the FA core complex genes. These motifs consisted of short sequences that are distributed throughout the entire tested region (Fig. 3A and B). However, in the 5′ region of the promoter sequences, these common motifs failed to follow a specific pattern. The 5′ region (approximately −1000 to −550) was found to harbor a more random distribution of the DNA motifs that did not contain transcription factor binding sites. In contrast, there were two specific pattern-forming sequence motifs that were clustered in the central portion of the promoter regions (positions −250 to −550). Fig. 3C depicts these motifs as sequence logos and shows the transcription factor binding sites within these sequence motifs. The sequence motif that is indicated by a purple bar includes one binding site each for E2F and TFII-I. The sequence represented by the green bar contains one binding site for E2F. There seemed to be no correlation between the numbers and types of the two DNA motifs and the promoter strength.

### Predicted transcription factor binding sites (TBSs)

**Figure 4:**
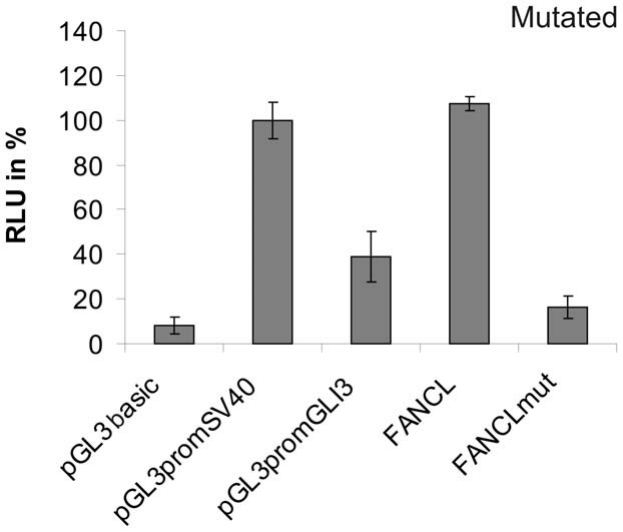
The promoter mutation in *FANCL*. The 219 bp deletion in the proximal region of the promoter led to a strong reduction in promoter activity.

Using *in silico* approaches (PROMO and the Genomatix suite), we identified a large number and variety of TBSs. Therefore, we wanted to identify the TBSs with different sets of consensus similarities: 70% and 85%. However, the overall set of TBSs was similar in all of the L1 promoter fragments. Some binding sites were shared by all of the FA core complex gene promoters, including those for the TFII-I, TFIID, E2F, STAT4, YY1, c-Jun and IRF1 proteins, and an additional group of shared, but not ubiquitously present, binding sites were found (Table 2). The important *cis*-regulatory elements were not distributed over the entire promoter region, but they were clustered within the portions with the highest transcriptional activity, including most of the E2F, YY1, STAT1, SMAD, AP1 and SP1/GC box *cis*-regulatory elements. The SMAD sites were not represented as often as the STAT or other sites in the 85% matrix similarity interval. However, we also focused on the SMAD sites, because of the data in the literature for FANCA [21] and crosstalk between STAT and SMAD [22]. The general importance of these TBSs was illustrated by a patient-derived *FANCL* genomic deletion that removes 219 bp of sequence upstream of the TSS and led to the loss of a region with a large number of TF recognition sites. This deletion resulted in a remarkable breakdown of transcription *in vitro* (Fig. 4).

**Table 2:** Transcription factor binding sites not common to all of the FA core complex genes.

FANCA	FANCB	FANCC	FANCE	FANCF	FANCG	FANCL	FANCM
STAT1beta	STAT1beta	STAT1beta	STAT1beta	STAT1beta	STAT1beta	STAT1beta	STAT1beta
SP1	SP1	SP1	SP1		SP1	SP1	
XCPE	XCPE	XCPE	XCPE		XCPE	XCPE	
AP1		AP1			AP1	AP1	
SMAD	SMAD^1^			SMAD^1^	SMAD	SMAD^1^	
GATA3^1^			GATA3^1^				
		STAT5A		STAT5A	STAT5A	STAT5A	STAT5A
		c-Fos					c-Fos
	NFκB^1^					NFκB^1^	

1 This factors were found only with a 70% sequence similarity.

### Confirmation of DNA-protein interactions

**Figure 5:**
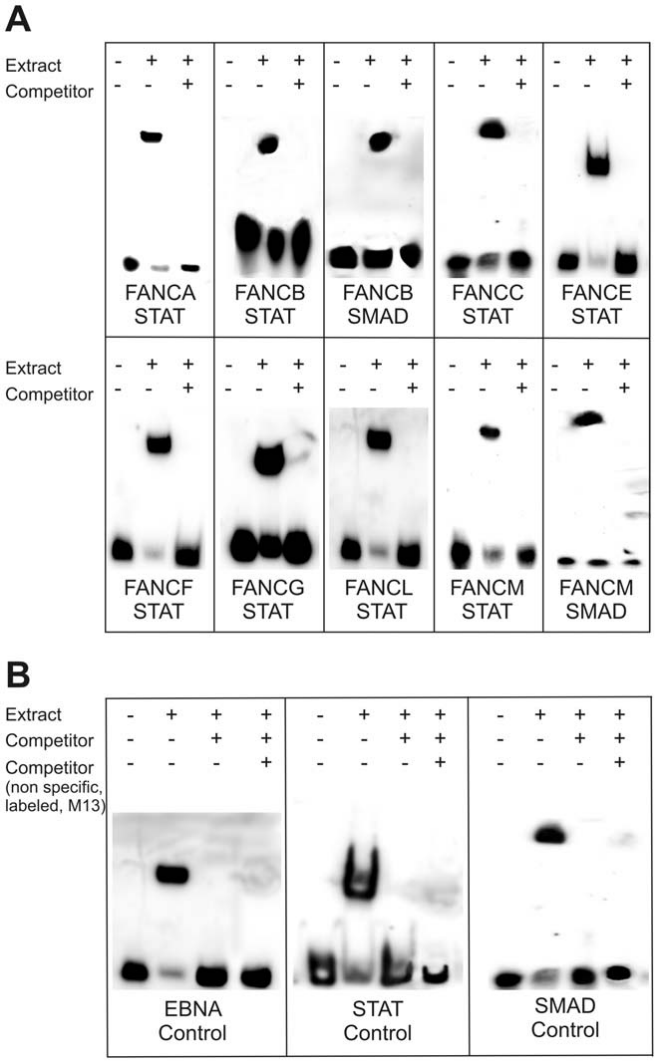
Electrophoretic mobility shift assay (EMSA) illustrating the DNA/protein interactions between the FA gene promoters and STAT/SMAD. (**A**) A positive result (band shift) was observed with all the tested FA genes. Lane 1: control, Epstein-Barr nuclear antigen (EBNA) extract. Lane 2: affirmed interactions. Lane 3: 200-fold molar excess of unlabeled DNA (cold competitor) as the control for specificity. (**B**) In these control reactions an additional non specific, but labeled oligo (*E.coli* M13) was added to show that a positive shift was not caused by the addition of any type of DNA (unspecific shift).

EMSA techniques were used to examine the DNA-protein interactions between the FA core complex promoters and the transcription factors that were predicted by the *in silico* tools. We focused on STAT1/4 and SMAD1/4 as the prominent members of known regulation-associated pathways [23], [24]. In each of the tested promoter sequences, we found either a STAT or a SMAD binding site. A prominent band shift was observed when the HeLa cell extract was incubated with the biotin-labeled oligonucleotide (Figure 5, lane 2). To prove the specificity of the observed interaction, we added a 200-fold molar excess of unlabeled, specific competitor sequences and the band shift was suppressed (Fig. 5, lane 3). Due to the binding of the specific, unlabeled competitor this band proved the specificity of the interaction in all experiments.

### The expression of FA core complex genes is influenced by STAT1/4 and SMAD1/4

**Figure 6:**
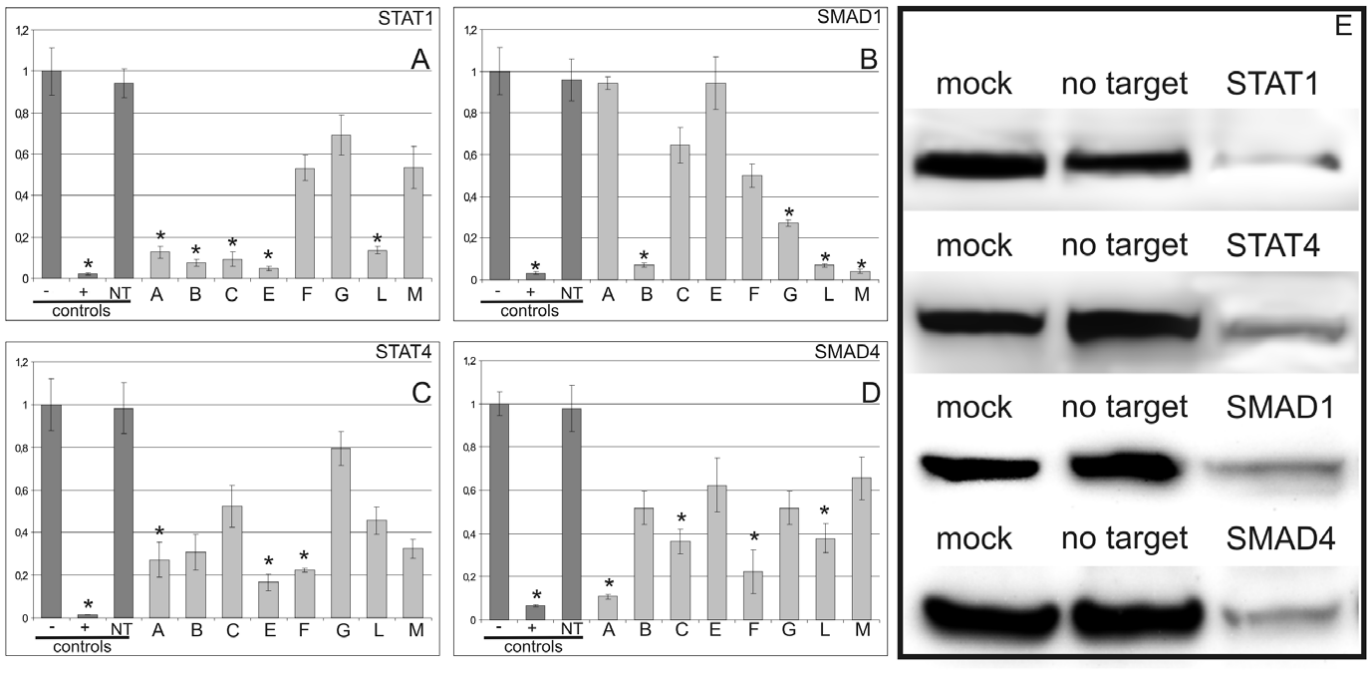
The consequences of STAT and SMAD downregulation on FA gene expression in HeLa cells. (**A**) The strongly reduced mRNA expression of *FANCA, -B, -C, -E and -L* following *STAT1* knockdown in the respective FANC gene (*FANCA*, *-B*, *-C*, *-E*, *-F*, *-G*, *-L* and *M* correspond to lanes 1–8, respectively). (**B**) The *SMAD1* knockdown results in decreased levels of *FANCB*, -*G*, *-L* and -*M* mRNAs. (**C**) The mRNA levels of *FANCA*, *-E* and *-F* were reduced following the siRNA treatment directed against *STAT4*. (**D**) Following the SMAD4 inactivation, *FANCA*, *-C*, *-F* and *-L* exhibited reduced mRNA levels. (**E**) A western blot showing the effective transcription factor knockdown. The first three columns denote the controls as follows: (−) untransfected HeLa cells, (+) knockdown using siRNA directed against the gene of interest, and (NT) transfection with a non-targeting siRNA. All the results were derived from triplicate assays. *p<0.05.

The results of our siRNA experiments support the notion of shared regulatory functions between the FA core complex gene promoters and prominent cellular pathways, such as TGF-β and JAK/STAT. Following siRNA knockdown of *STAT1/4* or *SMAD1/4*, we examined the mRNA expression of the FANC core complex genes. Knockdown of *STAT1* led to a significant decrease in the amount of detectable *FANCA*, *-B*, *-C*, *-E* and *-L* transcripts. Less than 15% of the normal level of transcript was present for these genes, which contain STAT1-responsive promoters (Fig. 6A). A similar, but slightly more variable outcome was obtained after *SMAD1* knockdown. The amount of significantly reduced FA gene transcripts (*FANCB, G, -L and -M*) varied from 27% to 3% of their original expression (Fig. 6B). These results indicate that two prominent members of the TGF-β and JAK/STAT pathways assume regulatory functions within the FA core complex gene promoters. A similar regulatory effect of *STAT4*, which is another member of the JAK/STAT pathway, was also observed (Fig. 6C). The siRNA induced downregulation of *STAT4* caused significant decreases in *FANCA*, -*E* and -*F* transcripts. The maximum transcript level for *FANCA* was 27% of the non-treated samples (*p<0.05*). The results of the *SMAD4* knockdown studies in the HeLa cells are of special note because a direct regulatory interaction between *SMAD4* and *FANCA* has been previously reported in mice [21].

Following *SMAD4* downregulation, *FANCA* showed the strongest decrease in expression, which was 10% (0.1-fold) of the original transcript level (Fig. 6D). *FANCC*, *-F* and *-L* were also downregulated, although less dramatically and with more variability (22%–37%). Control western blots showed an adequate knockdown of the transcription factors (Fig. 6E).

**Figure 7:**
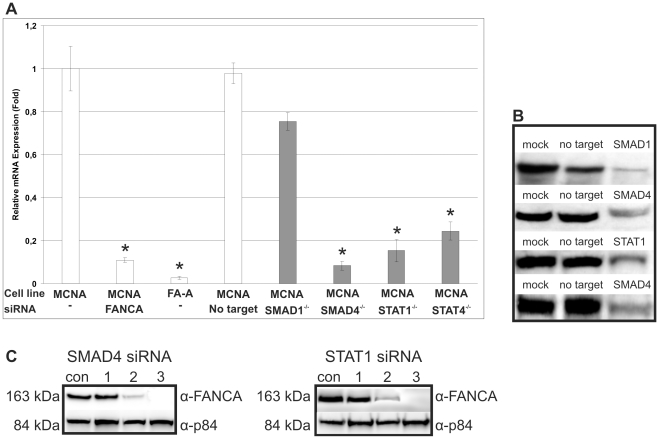
*FANCA* mRNA expression in normal control (MCNA) and FA-A (FA-A) primary human fibroblasts after siRNA treatment against SMAD1, SMAD4, STAT1 and STAT4. (**A**) Wild-type and FA-A controls (MCNA; FA-A) were left untreated. MCNA was also transfected with non-target siRNA. *FANCA* siRNA transfected con cells and patient-derived FA-A cells served as the positive controls. The results using primary fibroblast cell strains confirmed the results obtained in the HeLa cell system. (**B**) A western blot showing the effective transcription factor knockdown. (**C**) Depletion of FANCA protein was detected by an anti-FANCA antibody to determine the effectiveness of the knockdown at the protein level. Control untreated (lanes 1). SMAD4 and STAT1 siRNA-treated cells showed marginally detectable FANCA protein (lanes 2). *FANCA*-mutated (FA-A) cells are shown for comparison (lanes 3). The nuclear matrix protein (p84) served as a loading control.

To exclude cell specific effects, we repeated our knockdown experiments in a wild-type, primary human fibroblast cell strain (MCNA; con) and compared these results to patient-derived cultured fibroblasts carrying biallelic mutations in *FANCA* (MAKE; FA-A). Inactivating *SMAD4*, *STAT1* and *STAT4* caused *FANCA* downregulation in both the wild-type and the mutated cells (Fig. 7A). There was no response for SMAD1, and both of these findings were consistent with our previous experiments in HeLa cells. The western blots demonstrated an adequate knockdown of the transcription of these genes (Fig. 7B). Almost no visible FANCA protein was observed due to the *SMAD4/STAT1* knockdown (Fig. 7C, lane 2). Protein derived from a patient who belongs to the Fanconi anemia complementation group A (FA-A) is shown in lane 3 (Fig. 7C).

## Discussion

The aim of our study was to determine the structural and functional features of the regulatory machinery that drives the FA core complex genes. This included the common and rare transcription factor binding sites and their distribution. Similarities in all of the promoters might show a co-evolution in the promoters of FA core complex genes.

The cloned FA core complex gene promoter regions have characteristics that are typical of housekeeping genes. These characteristics include the following: a high GC content and CpG islands, a lack of obvious TATA or CCAAT regulatory sequences, and the presence of several transcription factor binding sites, including YY1, STAT1, AP2 and SP1/GC box *cis*-elements [19]. These properties suggest that the FA core complex gene promoters belong to a distinct subgroup of genes that are characterized by a dominant TSS surrounded by several weaker TSSs [17]. Our data showed that the region with the highest activity was situated within the central and proximal sections of the promoters, which is typically a high-activity region that has been described for a number of other genes [25], [26], [27]. Compared to the SV40 promoter, our L2 and L3 constructs showed comparable and sometimes higher activities, indicating that these regions harbor strong elements for the direction of the preinitiation complex. This was unexpected because the FA family of genes and their proteins are members of developmental and genome maintenance pathways that are similar to the GLI3 promoter and show mostly weak activity [20]. The lower level of activity that was observed with our L1 construct suggests the presence of a silencing element (or repressor) within this particular region. This is not without precedent because silencing elements have been identified in the human AZFa gene promoter [28], even if they are in a monodirectional position. The FA core complex genes are distributed independently and widely throughout the genome. With the exception of *FANCB*, these genes generally do not overlap with other genes on a large scale. In the present study, *FANCB* was the only gene with a slight bidirectional, basal promoter activity in the dual luciferase assay (Fig. 2A). This might be because *FANCB* harbors a promoter region that partially overlaps the human *MOSPD2* (motile sperm domain containing 2) gene. Cell-specific effects on the measurements were excluded because the results differed marginally between the cell lines.

The transcriptional regulation of genes that participate in protein complex formation is often marked by sequence similarities [29], [30]. Two different motifs were characterized within the most active part of the promoters that were specific to the FA core complex gene in this particular combination (Figs. 3B and C). With the exception of *FANCF*, all of the core complex gene promoters feature one or both of these two motifs, albeit in different numbers. Similar to what has been reported for *FANCD2*, the presence of binding sites for E2F in these motifs suggests that the E2F/Rb pathway also may be involved in regulating the FA core complex [31]. Furthermore the location of these motives in the 3′ region in combination with the silencing elements in the 5′ region underlined the bi-partite nature of these promoters.

Regulation of the JAK/STAT pathway by the FA/BRCA pathway has been noted previously [32]; however, in this study, we asked the opposite question, regulation of FA/BRCA by the JAK/STAT pathway. Our *in silico* data indicated that all of the FA core complex gene promoters featured one or more of the STAT1/4/5 binding motifs in combination with SP1, which is a cofactor for STAT1. Independent but closely spaced DNA binding sites for STAT and other transcription factors seem to be required for maximal transcriptional activity [33]. In contrast to the uniform presence of STAT binding sites, a SMAD binding site was detected only in the *FANCG* and *FANCA* promoters and, with a lower consensus conservation, in *FANCB*, *FANCF* and *FANCL*. This suggests that SMAD participates in a regulatory network that includes TGF-β [23], [34], [35]. To include SMAD in our analyses, we were encouraged by other studies that suggested that STAT and SMAD may form a complex and work cooperatively [36], [37]. A further demonstration of SMAD driven regulation is the AP1 and c-Jun/c-Fos binding sites that are present in most of the core complex FA gene promoters. The SMAD consensus motif, GTCTAGAC, is a palindromic sequence with two copies of GTCT, with its reverse compliment AGAC on the opposite strand. Tandem repeats of this sequence have been shown to promote TGF-β-inducible transcriptional activation [38]. SMAD also binds to TGAGTCAGAC, an AP1 binding site (TGAGTCA) that overlaps with an AGAC-containing SMAD-binding sequence [39]. A direct influence of SMAD4 on FA gene expression has been previously shown in mice [21] and was confirmed by the present study. Of note, loss of SMAD4 increases genomic instability and appears to contribute to the emergence of head and neck squamous cell carcinoma (HNSCC), which is a tumor that is frequently encountered in FA patients [21], [40].

There are only single reports concerning the possible cross-talk between two of the FANC core complex genes (*FANCA* and *FANCC*) and the TGF-β and JAK/STAT pathways [32], [41], [42]. Our present study confirms and extends these findings to all of the FA core complex members. To substantiate the results that were obtained with the HeLa cells, we repeated and confirmed the results of our experiments using normal (control) or FA-A primary fibroblast cultures.

The regulation of the FA core complex genes is unlikely to occur only through their promoters at the genomic level. Regulation at the transcript level has been recognized as an important aspect in the control of expression for many genes. In *Drosophila*, the box motifs “(the K box, the GY box and the Brd box)” appear to function as regulatory elements for the Notch signaling pathway [43], [44]. As mediators of miRNA binding, these three sequence motifs play key roles in regulating gene expression [43], [44], [45], [46]. Our analysis showed that K-boxes occur in most of the FA core complex transcripts, and at least one of these motifs can be predicted in each transcript, with the exception of *FANCE*.

Collectively, our study provides the first approximation of the features that govern the activity of the FA core complex group of genes. Based on the similarities, our analysis also adds support to the hypothesis of co-evolved promoters within this group. Given the importance of DNA damage recognition and repair for the prevention of premature cancer and aging, it seems reasonable for evolution to have provided additional protection for long-living, warm-blooded species. These protections include a certain degree of regulatory and functional redundancy, which was found in the FA core complex genes.

## Materials and Methods

The used fibroblast cell line MAKE derived from a Fanconi anemia patient, which is unknown to me, during the routine diagnostic process. The cell line was neither used for clinical studies nor for other studies and was processed anonymously. It is used only as a positive control cell line in complete accordance with the German gene diagnostic law that require the consent of the patient and/or their parents. For this type of use of patient material no approval of an ethics committee is required in German law. A written informed consent was obtained for research on this patient sample.

### PCR

Three products were amplified that were 5′ to the putative transcription start site (TSS; labeled L1, L2 and L3) for each of the core complex genes. L1, L2, and L3 were approximately 1 kb, 500 bp, and 200 bp upstream of the TSS, respectively. Primer sequences were generated using the Primer3 program (www.frodo.wi.mit.edu). Phusion DNA Polymerase (Finzymes) was used primarily, but the CG-rich PCR kit (Roche) was used for difficult templates, such as those with CG-rich regions. All the PCR experiments were performed in a volume of 50 µl and a primer concentration of 10 pmol. With the exception of the FAAP100 primers, all the primers were generated with a *KpnI* restriction site at the forward strand and a *HindIII* site at the reverse strand. For FAAP100, we used a *MluI* site on the forward strand because there was an internal *KpnI* site in the sequence. The GLI3 promoter is a reference promoter with relatively weak activity, and it was amplified with *KpnI/HindIII* restriction sites.

### Generation of plasmid constructs

The amplified PCR products were digested with their respective enzymes and ligated into the pGL3-Basic Vector (Promega), which contains the gene for firefly luciferase. To test the putative silencing properties of the 5′ FA promoter regions, we used the pGL3 SV40 vector. The constructs were transformed into competent *Escherichia coli* TOP10 (Invitrogen) or Turbo (NEB) cells according to the manufacturer's instructions. The integrity and direction of all the inserts were confirmed by sequence analyses using the RVprimer3 (forward) and the GLprimer2 (reverse) sequencing primers (Promega), which bind directly up- and downstream of the multiple cloning sites. Primer sequences are available upon request.

### Cell culture, transfection and dual luciferase assay

HeLa and HEK 293 cells (DSMZ-German Collection of Microorganisms and Cell Cultures) were grown in MEM containing 10% FCS (Gibco) and 1% penicillin/streptomycin (Gibco) under standard cell culture conditions (37°C and 5% CO_2_/95% air). The transfections were performed with Effectene transfection reagent (Qiagen) according to the manufacturer's protocol. Briefly, 24 h before transfection, cells were split into 12-well plates (200,000 cells/well) at a confluence level between 50 and 80%. The transfections required DNA (500 ng), the pGL basic vector (firefly) with our inserts, the pRL null vector (*renilla*) as an internal control, and 87 µl of transfection reagent (75 µl of EC Buffer, 6 µl of Enhancer and Effectene). At 48 h after transfection, the cells were washed with PBS and lysed with passive lysis buffer (Promega) to perform a conventional dual luciferase assay (DLR). Lysate (20 µl) was placed into each well of a white 96-well plate and measured with a Mithras Luminometer (Berthold Technologies). The DLR was performed according to the manufacturer's instructions, except that the amount of substrate was reduced to 50 µl per aliquot.

### Preparation of nuclear extracts

HeLa cells were used for the extraction of nuclear proteins using the NE-PER nuclear and cytoplasmic extraction reagents [47]. The cells were harvested with trypsin-EDTA and then centrifuged at 500× g for 5 minutes. The extraction steps were performed according to manufacturer's instructions. Nuclear extracts were stored at −80°C until use.

### Electrophoretic mobility shift assay (EMSA)

We performed a non radioactive, biotin labeled chemiluminescent EMSA [47]. We used oligonucleotides for the following binding reactions: *FANCA/*STAT, *FANCB/*STAT, *FANCB/*SMAD, *FANCC/*STAT, *FANCE/*STAT, *FANCF/*STAT, *FANCG/*STAT, *FANCL/*STAT, *FANCM/*STAT, and *FANCM/*SMAD. Binding reactions were performed for 20 min at room temperature in the presence of poly(dI-dC) (50 ng/µl), 0.05% Nonidet P-40, 5 mm MgCl_2_, 10 mm EDTA, and 2.5% glycerol in 1× binding buffer (LightShift chemiluminescent EMSA kit, Pierce) and biotin-end-labeled target DNA (20 fmol) and nuclear extract (4 µg). Unlabeled target DNA (4 pmol) was added per 20 µl of binding reaction where indicated. After a pre-electrophoretic run for 30 min at 100 V in 0.5× Tris borate/EDTA, aliquots were loaded onto 6% DNA retardation gels (Invitrogen) and electrophoresed for 50 min at 100 V. The gels were then transferred onto a positively charged nylon membrane (Nytran SPC, Whatman) in 0.5× Tris borate/EDTA at 380 mA for 50 min. The samples were cross-linked to the membrane for 15 min on a transilluminator equipped with 312 nm bulbs. Detection was performed using horseradish peroxidase-conjugated streptavidin in combination with the chemiluminescent substrate (LightShift chemiluminescent EMSA kit) according to the manufacturer's instructions.

### siRNA experiments

Knockdown experiments were performed in HeLa cells, non-FA human fibroblasts (ATCC; American Type Culture Collection) and in a patient derived, primary fibroblast cell lines that were either *FANCA* wild-type (MCNA; con) or which had compound, heterozygous *FANCA* mutations (MAKE; c.3349-1 G>A and c.4069 G>C; written and informed consent was obtained for this patient sample. For this type of use of patient material no approval of an ethics committee is required in German law). We used ON-TARGETplus siRNA (Dharmacon) against *STAT1*, *SMAD1*, *STAT4* and *SMAD4* and a DharmaFECT transfection reagent. Twenty-four h before transfection, the cells were split into 12-well plates (150,000 cells/well for HeLa cells and 120,000 cells/well for fibroblasts) at a confluence level between 50% and 80%. The transfections used siRNA solutions (5 µM) in 1× siRNA buffer (Dharmacon). The transfection reactions were performed according to the manufacturer's instructions. After 48 h, the cells were harvested for mRNA analyses.

### RNA extraction, cDNA synthesis and qRT-PCR analyses

Total RNA was obtained with the RNeasy Kit (Qiagen), and it was used for DNase digestion and cDNA synthesis. Up to 10 µg of RNA was treated with the TURBO DNA-free kit (Ambion) at 37°C for 30 min to remove contaminating DNA. Total RNA (1 µg), anchored oligo(dT)_18_ primer (2.5 µM) and reverse transcriptase (10 U) from the transcriptor high fidelity cDNA synthesis kit (Roche) were used for the first strand synthesis according to the manufacturer's suggestions. The cDNA was stored at −20°C.

The qRT-PCR primers for *STAT1*, *STAT4*, *SMAD1*, *SMAD4* and *FANCA* were determined using Primer Design™. Each of the samples was examined in triplicate and subjected to qRT-PCR using PerfeCTa SYBR Green SuperMix (Quanta). *GAPDH*, *ACTB* and *UBC* probes were used as internal controls. The relative RNA expression levels were determined by normalizing them to internal controls, and the values were calculated using the comparative Ct method. Statistical differences between 2 groups of data were analyzed using the 2-tailed Student's *t* test. *P* values less than 0.05 were considered significant.

### Western blot analysis

After the knockdown experiments were completed, immunoblots were performed with the samples containing total protein (40 µg) and 7% NuPage Tris-acetate polyacrylamide gels (Invitrogen). The membranes were probed with rabbit polyclonal anti-FANCA (1∶1000; Abcam ab5063). The secondary antibody was a horseradish peroxidase-linked donkey anti-rabbit IgG (1∶4000; GE Healthcare NA934V), and it was detected by the chemiluminescence technique using the ECL system (Amersham). For a loading control, we used a mouse monoclonal anti-p84 (nuclear matrix protein 84; 1∶2000; Abcam ab487). The secondary antibody was a horseradish peroxidase-linked goat anti-mouse IgG (1∶5000; Abcam; ab20043).

### Bioinformatics

The promoter regions were predicted *in silico* using the promoter prediction and gene2promoter programs (www.genomatix.de) and the UCSC genome browser. The TSSs were taken from the DTSS database where the TSS data were generated by massively sequencing the full-length cDNAs in humans and mice (www.dbtss.hgc.jp). The putative promoter sequences were analyzed for transcription factor binding sites with two software tools: Mat Inspector (Genomatix), and the PROMO tool/database with two sets of similarity (70% and 85%) (Transfac 8.3) (http://alggen.lsi.upc.es/cgi-in/promo_v3/promo/promoinit.cgi?dirDB=TF_8.3) [48], [49]. The MEME suite can be found under the following link: http://meme.sdsc.edu/meme4_5_0/intro.html.
